# Dislocation and Joint Fractures

**Published:** 1910-11

**Authors:** 


**Dislocation and Joint Fractures.** By **Frederick Jay Cotton,** A.M. M.D., First Assistant Surgeon, Boston City Hospital. Octavo of 654 pages, 1201 original illustrations. Philadelphia and London: W. B. Saunders Company. 1910. (Cloth, $6.00; half morocco, $7.00, net prices.)

Here is a new book and a good one. It is well written and well illustrated, the writer himself having been the artist. From the first page to the last the subject is dealt with in a most practical and able manner. The more we study fractures and luxations, the more apparent it becomes that each fracture is a mechanical problem in itself, and the treatment of lesions of this kind involves upon the part of the surgeon the responsibility of having his work tested by results, as well as intentions. Our duty is to obtain in a given case the best results possible by whatever means are at hand, often irrespective of traditional methods. The author's clinical and teaching experience has evidently fitted him to write a practical work and he has done it well. J. A. R.

